# Prognostic implication of *PD‐L1* polymorphisms in non‐small cell lung cancer treated with radiotherapy

**DOI:** 10.1002/cam4.4329

**Published:** 2021-10-06

**Authors:** Min Kyu Kang, Shin Yup Lee, Jin Eun Choi, Sook Kyung Do, Moon‐June Cho, Jun‐Sang Kim, Jae Yong Park

**Affiliations:** ^1^ Department of Radiation Oncology School of Medicine, Kyungpook National University Daegu South Korea; ^2^ Department of Internal Medicine School of Medicine, Kyungpook National University Daegu South Korea; ^3^ Department of Biochemistry and Cell Biology School of Medicine, Kyungpook National University Daegu South Korea; ^4^ Cell and Matrix Research Institute School of Medicine, Kyungpook National University Daegu South Korea; ^5^ Department of Radiation Oncology Chungnam National University School of Medicine Daejeon South Korea

**Keywords:** non‐small cell lung cancer, PD‐L1, polymorphisms, radiotherapy

## Abstract

**Background:**

To investigate the impact of *programmed death*‐*ligand 1 (PD*‐*L1)* polymorphisms on the prognosis of non‐small cell lung cancer (NSCLC) patients treated with curative radiotherapy.

**Methods:**

Four single nucleotide polymorphisms (SNPs) (rs822336G>C, rs822337T>A, rs822338C>T, and rs2297136A>G) in the *PD*‐*L1* gene were evaluated in 124 NSCLC patients. Clinical stage was I in 28, II in 17, and III in 79 patients. Fifty‐seven patients received radiotherapy alone, including 28 patients who received stereotactic body radiotherapy. Sixty‐seven patients received sequential or concurrent chemoradiotherapy. Risk factors for survival outcomes were analyzed with the log‐rank test and multivariate Cox proportional hazards models.

**Results:**

The rs822336GC+CC genotype was associated with better overall survival (OS) (hazard ratio [HR] = 0.60, 95% confidence interval [CI] = 0.37–0.97, *p* = 0.036) and regional failure‐free survival (RFFS) (HR = 0.32, 95% CI = 0.14–0.76, *p* = 0.009), compared with rs822336GG genotype. The rs822337TA+AA genotype was associated with better OS (HR =0.54, 95% CI = 0.34–0.88, *p* = 0.014), progression‐free survival (PFS) (HR = 0.64, 95% CI = 0.41–0.99, *p* = 0.046), and RFFS (HR = 0.38, 95% CI = 0.17–0.81, *p* = 0.013), compared with rs822337TT genotype. Three SNPs (rs822336, rs822337, and rs822338) were in linkage disequilibrium. Combined GTC and GTT (GT*) haplotype was associated with significantly worse OS (*p* = 0.018), PFS (*p* = 0.044), and RFFS (*p* = 0.038), compared with those with other combined haplotypes. Patients with diplotypes of two GT* haplotypes showed significantly worse OS (*p* = 0.023) and RFFS (*p* = 0.014) than those with other diplotypes.

**Conclusions:**

These findings suggest that *PD*‐*L1* polymorphisms could be predictive markers for NSCLC patients receiving radiotherapy.

## Graphical abstract

Genotypes of two SNPs (rs822336G>C and rs822337T>A) and the risk groups of diplotypes of rs822336‐rs822337‐rs822338 haplotypes in the *PD*‐*L1* gene were significantly associated with overall survival. These genotypes were related to regional failure‐free survival in terms of patterns of initial failure.

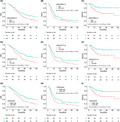

## 1 INTRODUCTION

Radiotherapy has been used to treat early to advanced non‐small cell lung cancer (NSCLC) with a curative intent. To be brief, while radiotherapy can be an alternative to surgery for early stage NSCLC patients who cannot undergo surgery for any reason, radiotherapy combined with chemotherapy is recommended for locally advanced NSCLC patients. Recently, a randomized controlled trial revealed that consolidation immunotherapy after concurrent chemoradiotherapy increased overall survival (OS) of locally advanced NSCLC patients.^1^, ^2^

Along with the growing interest in immune checkpoint inhibitors in cancer treatment, many investigations in the field of radiotherapy have focused on programmed death‐ligand 1 (PD‐L1) protein which plays an important role for cancer cells to escape immune surveillance.^3^ Some researchers explored the impact of baseline PD‐L1 expression on post‐radiotherapy outcomes in NSCLC, but the results were contradictory.^4^, ^5^ Others reported that changes in PD‐L1 expression and density of CD8+ tumor‐infiltrating lymphocytes after radiotherapy were related to the prognosis of NSCLC patients treated with preoperative concurrent chemoradiotherapy, with no association between the baseline PD‐L1 status and changes after radiotherapy.^6^, ^7^ Considering that upregulation of PD‐L1 expression after irradiation led to radioresistance in animal tumor models,^8^, ^9^ it can be presumed that the capability of PD‐L1 expression after radiotherapy would be important to determine the prognosis, rather than the baseline status. PD‐L1 is encoded by the *PD*‐*L1* gene located on chromosome 9 at position p24.1, whose polymorphisms have been reported to be predictive markers in NSCLC patients who received chemotherapy or surgery.^10^, ^11^, ^12^, ^13^ However, there has been no report about the influence of *PD*‐*L1* polymorphisms on the post‐radiotherapy prognosis in any type of cancer.

Therefore, we hypothesized that *PD*‐*L1* polymorphisms may affect the prognosis of NSCLC patients receiving radiotherapy. To examine this hypothesis, we examined the relationship between *PD*‐*L1* polymorphisms and treatment outcomes in NSCLC patients treated with radiotherapy.

## 2 MATERIALS AND METHODS

### 2.1 Patients

**FIGURE 1:**
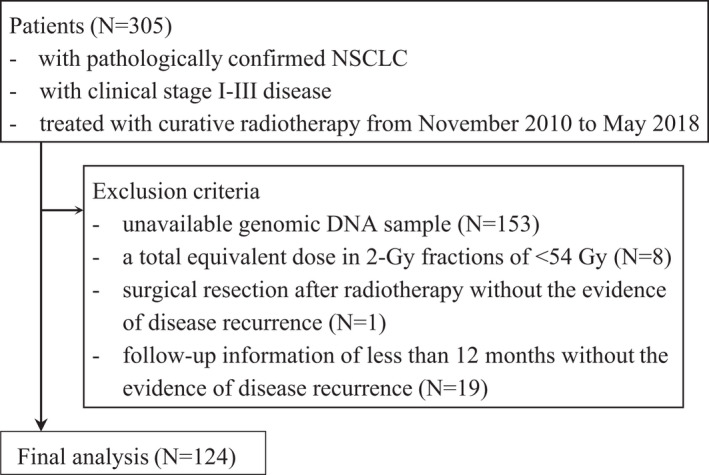
Flowchart of the study population

From November 2010 to May 2018, 305 patients with pathologically confirmed clinical stage I‐III NSCLC were treated with curative radiotherapy in our institution. Clinical TNM stage was evaluated according to the AJCC 8th staging system.^14^ Of them, 152 patients had available genomic DNA samples for single nucleotide polymorphisms (SNPs) genotyping. After excluding the patients who received a total equivalent dose in 2 Gy fractions of less than 54 Gy (*N* = 8), undertook surgical resection after radiotherapy without the evidence of disease recurrence (*N* = 1), or had follow‐up information of less than 12 months without the evidence of disease recurrence (*N* = 19), this study enrolled 124 patients for analyses (Figure 1). The study was conducted according to the guidelines of the Declaration of Helsinki, and approved by the Institutional Review Board of the Kyungpook National University Chilgok Hospital (2019‐01‐025). The need for informed consent was waived in consideration of the retrospective study design.

### 2.2 SNP selection and genotyping

Among five *PD*‐*L1* SNPs which were selected in a previous study,^12^ four SNPs (rs822336G>C, rs822337T>A, rs822338C>T, and rs2297136A>G), which were applicable to the iPLEX^®^ Assay and MassARRAY^®^ System (Agena Bioscience), were genotyped. The linkage disequilibrium (LD) status was determined with Haploview ver. 4.2 software.^15^ Then, the haplotype frequencies were estimated using the Phase ver. 2.1.1 software package.^16^

### 2.3 Statistical analysis

The distribution of clinicopathologic factors according to genotype was compared with Pearson's chi‐square test, Fisher's exact test, Student's *t*‐test, and Mann–Whitney *U*‐test as appropriate. Survival rates were estimated from the first day of treatment to the date of the event or the last follow‐up with the Kaplan–Meier method: overall survival (OS), progression‐free survival (PFS), local failure‐free survival (LFFS), regional failure‐free survival (RFFS), and distant metastasis‐free survival (DMFS). Primary patterns of failure were used to calculate LFFS, RFFS, and DMFS, while considering failures with an interval of 3 months or less as simultaneous events. Risk factors for survival outcomes were analyzed with the log‐rank test and multivariate Cox proportional hazards models. R statistics (ver. 4.0.3, The R Foundation for Statistical Computing, Vienna, Austria) were used for statistical analyses. Values of *p* <0.05 were considered statistically significant.

## 3 RESULTS

### 3.1 Characteristics

Patient and tumor characteristics are shown in Table 1. The median age was 70 years (range: 45–87); 107 patients were male. TNM stage was I in 28, II in 17, and III in 79 patients. Twenty‐eight patients with cT1‐4N0 received stereotactic body radiotherapy (SBRT) without any adjuvant treatment. Ninety‐six patients with stage I–III received intensity‐modulated radiotherapy or three‐dimensional conformal radiotherapy (referred to as the non‐SBRT subgroup). Combination therapy in the non‐SBRT subgroup was radiotherapy alone in 29, sequential chemoradiotherapy in 24, and concurrent chemoradiotherapy in 43 patients. The most common chemotherapy regimen for sequential or concurrent chemoradiotherapy was paclitaxel–cisplatin doublet. The details of radiotherapy and chemotherapy are summarized in Table S1. None of the patients received immune checkpoint inhibitors after radiotherapy without evidence of recurrence.

**TABLE 1:** Patient and tumor characteristics

	Patients
Age	
≤70 years	63 (50.8%)
>70 years	61 (49.2%)
Sex	
Male	107 (86.3%)
Female	17 (13.7%)
Histology	
Squamous cell carcinoma	75 (60.5%)
Adenocarcinoma	34 (27.4%)
Large cell carcinoma	1 (0.8%)
Non‐small cell carcinoma	14 (11.3%)
T stage	
x	1 (0.8%)
1	34 (27.4%)
2	40 (32.3%)
3	25 (20.2%)
4	24 (19.4%)
N stage	
0	44 (35.5%)
1	13 (10.5%)
2	44 (35.5%)
3	23 (18.5%)
TNM stage	
I	28 (22.6%)
II	17 (13.7%)
III	79 (63.7%)
Radiotherapy technique	
Stereotactic body radiotherapy	28 (22.6%)
Three‐dimensional conformal radiotherapy	69 (55.6%)
Intensity‐modulated radiotherapy	27 (21.8%)

### 3.2 Clinical factors and outcomes

With a median follow‐up of 29 (range: 4–116) months, OS, PFS, LFFS, RFFS, and DMFS rates of all patients were 58.9%, 29.4%, 57.8%, 66.4%, and 56.7% at 2 years, respectively. In all patients, age (≤70 years vs. >70 years), sex, TNM stage (I‐II vs. III), histologic type (adenocarcinoma vs. others), and radiotherapy modality (SBRT vs. non‐SBRT) were significant risk factors for at least one of the survival outcomes in the univariate analyses (Table S2). Age and sex were significantly associated with RFFS; TNM stage with OS, PFS, RFFS, and DMFS; histologic type with OS and LFFS; and radiotherapy modality with PFS, LFFS, and DMFS. Chemotherapy was not associated with any of the survival outcomes.

### 3.3 Allele frequencies of *PD*‐*L1* SNPs

The frequencies of the four SNPs are shown in Table 2. The distribution of clinical factors including sex, age, TNM stage, histologic type, total radiation dose, radiotherapy modality, and chemotherapy was not related to any of the four SNPs, except total radiation dose for rs822337 (data not shown).

**TABLE 2:** Profiles of four SNPs of the *PD*‐*L1* gene

	Location	CR	MAF	HWE‐p	wild/wild	wild/variant	variant/variant
rs822336G>C	Promoter	98.4%	0.23	0.420	74 (61%)	40 (33%)	8 (7%)
rs822337T>A	Promoter	96.8%	0.29	0.217	63 (53%)	44 (37%)	13 (11%)
rs822338C>T	Intron	100%	0.36	0.154	54 (44%)	50 (40%)	20 (16%)
rs2297136A>G	3′UTR	100%	0.20	0.592	80 (65%)	38 (31%)	6 (5%)

Abbreviations: CR, call rate; HWE‐p, p‐value for Hardy–Weinberg equilibrium; MAF, minor allele frequency.

Among the four SNPs, three SNPs (rs822336, rs822337, and rs822338) were in LD (|D′| = 1.0 and *r^2^* = 0.72 between rs8222336 and rs822337, |D′| = 1.0 and *r^2^* = 0.52 between rs8222336 and rs822338, and |D′| = 1.0 and *r^2^* = 0.74 between rs8222337 and rs822338). The most common haplotype and diplotype were GTC (63.74%) and GTC/GTC (43.5%), respectively. Table S3 shows the frequencies of haplotypes and diplotypes of the three SNPs.

### 3.4 *PD‐L1* polymorphisms and outcomes

**FIGURE 2:**
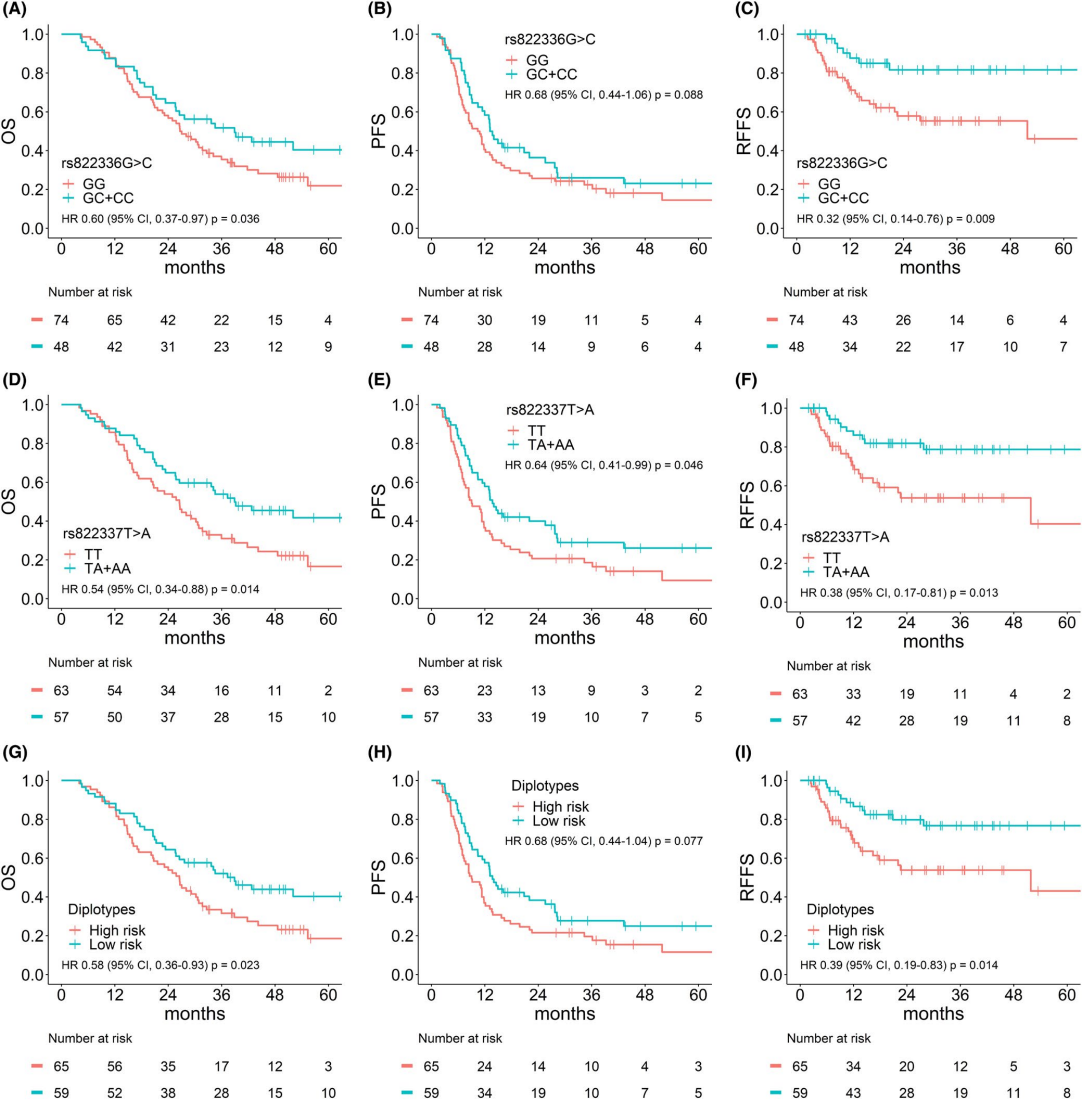
The survival curves according to the genotypes of rs822336 (A–C) and rs822337 (D–F) and the risk groups of diplotypes of rs822336‐rs822337‐rs822338 haplotypes (G–I). *p*‐values are from the multivariate Cox proportional hazards model. OS, Overall survival; PFS, progression‐free survival; RFFS, regional failure‐free survival

In the multivariate analyses adjusted for age, sex, TNM stage, tumor histology, radiotherapy modality, and chemotherapy, rs822336 and rs822337 were significantly related to outcomes (Table 3 and Table S4). The rs822336GC+CC genotype was associated with better OS (hazard ratio [HR] = 0.60, 95% confidence interval [CI] 0.37–0.97, *p* = 0.036) and RFFS (HR = 0.32, 95% CI = 0.14–0.76, *p* = 0.009), compared with the rs822336GG genotype (Figure 2A–C). The rs822337TA+AA genotype was related to better OS (HR = 0.54, 95% CI = 0.34–0.88, *p* = 0.014), PFS (HR = 0.64, 95% CI = 0.41–0.99, *p* = 0.046), and RFFS (HR = 0.38, 95% CI = 0.17–0.81, *p* = 0.013), compared with the rs822337TT genotype (Figure 2D–F).

**TABLE 3:** Multivariate analyses of four SNPs for treatment outcomes in all 124 patients in dominant models

	Hazard ratio (95% confidence interval)	*p*
Overall survival		
rs822336	0.60 (0.37–0.97)	0.036
rs822337	0.54 (0.34–0.88)	0.014
rs822338	0.69 (0.44–1.08)	0.102
rs2297136	0.82 (0.51–1.32)	0.417
Progression‐free survival		
rs822336	0.68 (0.44–1.06)	0.088
rs822337	0.64 (0.41–0.99)	0.046
rs822338	0.77 (0.51–1.18)	0.233
rs2297136	0.90 (0.58–1.39)	0.623
Local failure‐free survival		
rs822336	0.65 (0.36–1.20)	0.168
rs822337	0.75 (0.42–1.36)	0.349
rs822338	1.06 (0.60–1.87)	0.851
rs2297136	1.07 (0.59–1.94)	0.816
Regional failure‐free survival		
rs822336	0.32 (0.14–0.76)	0.009
rs822337	0.38 (0.17–0.81)	0.013
rs822338	0.57 (0.29–1.12)	0.101
rs2297136	0.94 (0.47–1.91)	0.869
Distant metastasis‐free survival		
rs822336	0.73 (0.41–1.31)	0.291
rs822337	0.83 (0.46–1.49)	0.529
rs822338	0.72 (0.41–1.27)	0.255
rs2297136	1.09 (0.61–1.94)	0.775

Note

All the results were from multivariate analyses adjusted for sex, age, TNM stage, histologic type, radiotherapy modality, and the use of chemotherapy.

As for the haplotypes of rs822336G>C‐rs822337T>A‐rs822338C>T, the combined GTC and GTT (GT*) haplotype was related to worse OS (*p* = 0.018), PFS (*p* = 0.044), and RFFS (*p* = 0.038), compared with those with combined other haplotypes (Table 4). GT* were defined as bad haplotypes, while others were defined as good haplotypes. Patients with at least one of the good haplotypes showed better OS (HR = 0.58, 95% CI = 0.36–0.93, *p* = 0.023) and RFFS (HR = 0.39, 95% CI = 0.19–0.83, *p* = 0.014) than those with two bad haplotypes (Table 4). Thus, diplotypes with two GT* haplotypes, GTC/GTC, and GTC/GTT, were classified as high‐risk diplotypes (vs. low‐risk diplotypes for others). Survival curves according to the risk groups of diplotypes are presented in Figure 2G–I.

**TABLE 4:** Multivariate analyses of haplotypes and diplotypes of rs822336G>C‐ rs822337T>C‐rs822338C>T in the *PD*‐*L1* gene

	Patients	OS		PFS		LFFS		RFFS		DMFS	
		HR (95% CI)	*p*	HR (95% CI)	*p*	HR (95% CI)	*p*	HR (95% CI)	*p*	HR (95% CI)	*p*
Haplotypes											
GTC	158	1.00		1.00		1.00		1.00		1.00	
GTT	18	1.26 (0.72–2.23)	0.419	1.49 (0.87–2.55)	0.151	1.66 (0.82–3.39)	0.161	1.05 (0.41–2.66)	0.921	1.14 (0.54–2.40)	0.734
GAT	15	0.63 (0.30–1.30)	0.211	0.76 (0.39–1.49)	0.429	1.51 (0.70–3.25)	0.290	0.65 (0.23–1.84)	0.415	1.12 (0.51–2.48)	0.778
CAT	57	0.67 (0.45–0.99)	0.045	0.73 (0.51–1.04)	0.083	0.72 (0.44–1.20)	0.206	0.51 (0.26–0.99)	0.046	0.83 (0.51–1.33)	0.435
*p*_trend_		0.034		0.084		0.363		0.040		0.500	
GT*	176	1.00		1.00		1.00		1.00		1.00	
Others	72	0.64 (0.45–0.93)	0.018	0.71 (0.51–0.99)	0.044	0.81 (0.52–1.26)	0.351	0.54 (0.30–0.97)	0.038	0.87 (0.56–1.34)	0.518
Diplotypes											
htB/htB	65	1.00		1.00		1.00		1.00		1.00	
htB/htG	46	0.62 (0.37–1.02)	0.062	0.73 (0.46–1.17)	0.190	0.77 (0.41–1.46)	0.427	0.34 (0.14–0.81)	0.015	0.93 (0.50–1.72)	0.821
htG/htG	13	0.49 (0.23–1.07)	0.074	0.55 (0.27–1.11)	0.093	0.72 (0.29–1.82)	0.491	0.54 (0.18–1.66)	0.282	0.74 (0.30–1.85)	0.520
*p*_trend_		0.025		0.057		0.375		0.047		0.541	
htB/htB	65	1.00		1.00		1.00		1.00		1.00	
htB/htG+htG/htG	59	0.58 (0.36–0.93)	0.023	0.68 (0.44–1.04)	0.077	0.76 (0.43–1.36)	0.354	0.39 (0.19–0.83)	0.014	0.88 (0.50–1.55)	0.654

Note

All the results were from multivariate analyses adjusted for sex, age, TNM stage, histologic type, radiotherapy modality, and the use of chemotherapy. Haplotypes of GT* (GTC and GTT) were defined as bad haplotypes (htB), while others (GAT and CAT) were defined as good haplotypes (htG).

Abbreviations: CI, confidence interval; DMFS, distant metastasis‐free survival; HR, hazard ratio; LFFS, local failure‐free survival; OS, overall survival; PFS, progression‐free survival; RFFS, regional failure‐free survival.

### 3.5 Subgroup analyses

The differences in survival outcomes between the risk groups of diplotypes were analyzed in the SBRT and non‐SBRT subgroups, respectively. In the SBRT subgroup, the patients with high‐risk diplotypes showed significantly worse PFS and RFFS in the multivariate analyses adjusted for sex, age, histologic type, and cT stage, with a tendency toward worse DMFS (Figure S1). In the non‐SBRT subgroup, high‐risk diplotypes had borderline significance for OS and RFFS in the multivariate analyses adjusted for sex, age, TNM stage, histologic type, and use of chemotherapy (Figure S2).

## 4 DISCUSSION

This study investigated whether *PD*‐*L1* polymorphisms could predict the prognosis in NSCLC patients treated with radiotherapy. Among four SNPs evaluated, rs822336 and rs822337 were significantly related to treatment outcomes. In diplotype analyses of the three SNPs with LD (rs822336, rs822337, and rs822338), the patients with high‐risk diplotypes showed significantly worse OS and RFFS than those with other diplotypes. These findings imply that the *PD*‐*L1* polymorphisms could be utilized as predictive markers for NSCLC patients receiving radiotherapy.

Currently, immunogenic cell death is considered an important mechanism of tumor cell death after radiotherapy, in addition to direct DNA damage.^3^, ^17^, ^18^ Damage‐associated molecular patterns released by irradiation activate dendritic cells, presenting tumor neoantigens, and activating CD8+ T cells.^18^, ^19^, ^20^ Radiotherapy also promotes T‐cell infiltration into tumors by upregulating the expression of adhesion molecules on endothelial cells and the release of cytokines.^21^ The activated CD8+ T cells are known to be important to reduce or eradicate the primary tumor, or distant metastasis after radiotherapy.^18^, ^22^ However, IFNγ produced by CD8+ T cells after radiotherapy can upregulate PD‐L1 expression on tumor cells, which in turn leads to radioresistance.^8^, ^9^

The programmed cell death 1 (PD‐1)/PD‐L1 axis has an important role in immune evasion of tumor cells.^3^ PD‐L1 expressed on tumor cells binds to PD‐1 on effector T cells, resulting in suppressing the cytotoxic activity of T cells.^23^ However, the significance of the baseline expression of PD‐L1 remains controversial in NSCLC patients.^4^ PD‐L1 expression at baseline has been reported to be either associated with no prognostic significance, better prognosis, or worse prognosis after surgery, chemotherapy, or radiotherapy.^7^, ^11^, ^12^, ^24^, ^25^, ^26^ In addition, the clinical importance of radiation‐induced upregulation of PD‐L1 expression is controversial, even though the expression of PD‐L1 has been reported to increase after radiotherapy in patients with various tumors including NSCLC.^6^, ^7^, ^27^, ^28^, ^29^ In soft tissue sarcoma, the rate of positive PD‐L1 expression (>1%) in tumor cells and tumor‐associated macrophages increased after preoperative radiotherapy, and positive PD‐L1 expression on tumor‐associated macrophages was significantly related to worse DMFS.^28^ In cervical cancer patients, patients with positive PD‐L1 expression (≥1%) after 12 Gy of carbon‐ion radiotherapy showed a significantly better PFS compared to those without PD‐L1 expression.^29^ In addition, the PD‐L1 expression level after preoperative chemoradiotherapy (≥50% vs. <50%) was not associated with OS after surgery in NSCLC.^7^

In the current study, the rs822336GG genotype, rs822337TT genotype, and high‐risk diplotypes of rs822336‐rs822337‐rs822338 in the *PD*‐*L1* gene were significantly related to worse OS and RFFS. An rs822336G‐rs822337T haplotype of the *PD*‐*L1* gene was reported to show a significantly increased promoter activity than an rs822336C‐rs822337A haplotype in a luciferase assay, suggesting rs822336G‐rs822337T is associated with an increased PD‐L1 expression.^12^ The poor prognosis of our patients with high‐risk diplotypes might be caused by having a pair of the rs822336G‐rs822337T haplotype. Fujimoto et al.^6^ revealed that NSCLC patients with increased PD‐L1 expression after preoperative chemoradiotherapy showed a significantly worse OS than those with unchanged or decreased PD‐L1 expression. Therefore, further studies are needed to investigate the relationship between the genotypes of the three SNPs and PD‐L1 expression, especially radiotherapy‐induced expression level change, along with the prognostic significance. It would be worthy to investigate whether *PD*‐*L1* polymorphisms may be utilized in identifying patients who would benefit from the combination of radiotherapy and PD‐1/PD‐L1 inhibitors.

This study has some limitations as a retrospective study. This study enrolled a relatively small number of patients, whose stages and treatment modalities were various. The status of PD‐L1 expression at baseline was not tested in most patients. However, the consistent significance of the effect of PD‐L1 genotypes on the prognosis, regardless of patient‐, tumor‐, and treatment‐related factors, could support the reliability of our results. In particular, the patients with high‐risk diplotypes experienced significantly poorer regional control in both the SBRT and non‐SBRT subgroups.

In summary, our results suggest that *PD*‐*L1* polymorphisms could be predictive markers for NSCLC patients receiving radiotherapy. As far as we know, this is the first study to report the prognostic value of *PD*‐*L1* polymorphisms in NSCLC patients treated with radiotherapy. Further studies are required to confirm our findings and to investigate the possible mechanisms in the relationship between *PD*‐*L1* polymorphisms and failures after radiotherapy.

## ETHICAL APPROVAL STATEMENT

The study was approved by the Institutional Review Board of the Kyungpook National University Chilgok Hospital (2019–01–025).

## CONFLICT OF INTEREST

The authors declare no conflict of interest.

## Supporting information

Supplementary Material

## Data Availability

The data that support the findings of this study are available from the corresponding author upon reasonable request.
