# Supplementary material for: Prognostic implication of PD‐L1 polymorphisms in non‐small cell lung cancer treated with radiotherapy
Source: Cancer Med. 2021 Oct 6;10(22):8071–8. doi: 10.1002/cam4.4329 (PMC8607250; doi:10.1002/cam4.4329)

### **Table S1.** Schedules of radiotherapy and chemotherapy.

|  | Fractionations | EQD2 (Gy) | Patients | Chemotherapy |
| --- | --- | --- | --- | --- |
| Stereotactic body radiotherapy  (N = 28) | 10 Gy x 5  7.5 Gy x 8  12 Gy x 4  12.5 Gy x 4  15 Gy x 4  18 Gy x 3 | 83.3  87.5  88.0  93.8  125.0  126.0 | 2  1  12  6  1  6 | No (28) |
| Conventional radiotherapy  (N = 84) | 1.8 Gy x 30  1.8 Gy x 35  2 Gy x 30  2 Gy x 32  2 Gy x 33  2 Gy x 35 | 53.1  62.0  60.0  64.0  66.0  70.0 | 1  8  4  1  66  4 | No (18)  Sequential (23)  Concurrent (43) |
| Hypofractionated radiotherapy  (N = 12) | 3 Gy x 18  3 Gy x 20  2.5 Gy x 25  2.2 Gy x 30  2.2 Gy x 31  2.2 Gy x 32  6 Gy x 10 | 58.5  65.0  65.1  67.1  69.3  71.6  80.0 | 2  2  1  2  3  1  1 | No (11)  Sequential (1) |

EQD2, equivalent dose in 2-Gy fractions.

###

### **Table S2.** Univariate analyses of clinical risk factors for survival outcomes in all 124 patients.

|  |  | Patients | OS | | PFS | | LFFS | | RFFS | | DMFS | |
| --- | --- | --- | --- | --- | --- | --- | --- | --- | --- | --- | --- | --- |
|  |  |  | 2YSR | p | 2YSR | p | 2YSR | p | 2YSR | p | 2YSR | p |
| Age | ≤70 years | 63 (50.8%) | 60.3% | 0.395 | 19.6% | 0.144 | 50.7% | 0.885 | 55.2% | 0.018 | 48.4% | 0.118 |
|  | >70 years | 61 (49.2%) | 57.4% |  | 39.2% |  | 64.5% |  | 78.4% |  | 65.7% |  |
| Sex | male | 107 (86.3%) | 54.2% | 0.077 | 30.3% | 0.586 | 54.5% | 0.062 | 69.1% | 0.046 | 56.5% | 0.996 |
|  | female | 17 (13.7%) | 88.2% |  | 23.5% |  | 77.1% |  | 52.9% |  | 57.8% |  |
| TNM stage | I-II | 45 (36.3%) | 75.6% | 0.015 | 55.2% | 0.001 | 77.6% | 0.077 | 79.6% | 0.042 | 78.4% | 0.003 |
|  | III | 79 (63.7%) | 49.4% |  | 15.2% |  | 45.0% |  | 58.4% |  | 44.2% |  |
| Histologic type | adenocarcinoma | 34 (27.4%) | 88.2% | 0.031 | 26.5% | 0.927 | 72.9% | 0.034 | 67.9% | 0.942 | 48.8% | 0.535 |
|  | others | 90 (72.6%) | 47.8% |  | 30.6% |  | 52.0% |  | 67.0% |  | 60.7% |  |
| Radiotherapy | SBRT | 28 (22.6%) | 71.4% | 0.077 | 63.3% | 0.002 | 86.4% | 0.018 | 82.4% | 0.125 | 80.0% | 0.029 |
|  | non-SBRT | 96 (77.4%) | 55.2% |  | 19.8% |  | 49.6% |  | 61.7% |  | 49.9% |  |
| Chemotherapy | no | 57 (46.0%) | 59.6% | 0.876 | 41.7% | 0.295 | 65.0% | 0.689 | 77.4% | 0.099 | 65.8% | 0.167 |
|  | yes | 67 (54.0%) | 58.2% |  | 19.4% |  | 51.2% |  | 57.8% |  | 49.7% |  |

OS, overall survival; PFS, progression-free survival; LFFS, local failure-free survival; RFFS, regional failure-free survival; DMFS, distant metastasis-free survival; 2YRS, 2-year survival rate; SBRT, stereotactic body radiotherapy.

### **Table S3.** Frequencies of haplotypes and diplotypes of rs822336G>C, rs822337T>A, and rs822338C>T in all 124 patients.

|  | Genotypes | Frequencies |
| --- | --- | --- |
| Haplotypes | GTC | 176 (63.7%) |
|  | GTT | 18 (7.3%) |
|  | GAT | 15 (6.0%) |
|  | CAT | 57 (23.0%) |
| Diplotypes | GTC/GTC | 54 (43.5%) |
|  | GTC/GTT | 11 (8.9%) |
|  | GTC/GAT | 9 (7.3%) |
|  | GTC/CAT | 30 (24.2%) |
|  | GTT/GAT | 1 (0.8%) |
|  | GTT/CAT | 6 (4.8%) |
|  | GAT/CAT | 5 (4.0%) |
|  | CAT/CAT | 8 (6.5%) |

### **Table S4.** Multivariate analyses of four SNPs for survival outcomes in all 124 patients.

|  | Hazard ratio (95% confidence interval), p-value | |
| --- | --- | --- |
|  | Codominant model | Recessive model |
| Overall survival | | |
| rs822336 | 0.68 (0.46–1.02), 0.061 | 0.77 (0.31–1.93), 0.574 |
| rs822337 | 0.65 (0.45–0.93), 0.018 | 0.61 (0.29–1.29), 0.192 |
| rs822338 | 0.77 (0.56–1.06), 0.111 | 0.74 (0.39–1.39), 0.347 |
| rs2297136 | 0.78 (0.54–1.15), 0.209 | 0.39 (0.11–1.31), 0.126 |
| Progression-free survival | | |
| rs822336 | 0.74 (0.52–1.04), 0.085 | 0.66 (0.29–1.53), 0.334 |
| rs822337 | 0.71 (0.52–0.98), 0.037 | 0.60 (0.30–1.20), 0.148 |
| rs822338 | 0.71 (0.52–0.98), 0.293 | 0.87 (0.50–1.52), 0.627 |
| rs2297136 | 0.86 (0.61–1.23), 0.406 | 0.56 (0.20–1.57), 0.274 |
| Local failure-free survival | | |
| rs822336 | 0.67 (0.41–1.11), 0.120 | 0.45 (0.11–1.89), 0.272 |
| rs822337 | 0.83 (0.54–1.27), 0.392 | 0.83 (0.34–2.04), 0.686 |
| rs822338 | 0.83 (0.54–1.27), 0.897 | 0.82 (0.38–1.79), 0.621 |
| rs2297136 | 0.92 (0.59–1.45), 0.727 | 0.42 (0.10–1.76), 0.233 |
| Regional failure-free survival | | |
| rs822336 | 0.51 (0.26–0.99), 0.048 | 1.16 (0.35–3.86), 0.808 |
| rs822337 | 0.56 (0.32–1.00), 0.052 | 0.82 (0.28–2.45), 0.725 |
| rs822338 | 0.56 (0.32–1.00), 0.095 | 0.55 (0.19–1.61), 0.275 |
| rs2297136 | 0.98 (0.55–1.76), 0.955 | 1.18 (0.27–5.14), 0.830 |
| Distant metastasis-free survival | | |
| rs822336 | 0.80 (0.51–1.27), 0.342 | 0.84 (0.30–2.37), 0.737 |
| rs822337 | 0.85 (0.56–1.28), 0.430 | 0.72 (0.30–1.75), 0.474 |
| rs822338 | 0.85 (0.56–1.28), 0.707 | 1.31 (0.66–2.60), 0.445 |
| rs2297136 | 1.01 (0.63–1.61), 0.970 | 0.70 (0.17–2.92), 0.619 |

*Note:* All the results were from multivariate analyses adjusted for sex, age, histologic type, TNM stage, radiotherapy modality, and the use of chemotherapy.

### **Figure S1.** Survival curves according to the risk groups of diplotypes of rs822336G>C-rs822337T>A-rs822338C>T in 28 patients treated with SBRT.

(A)
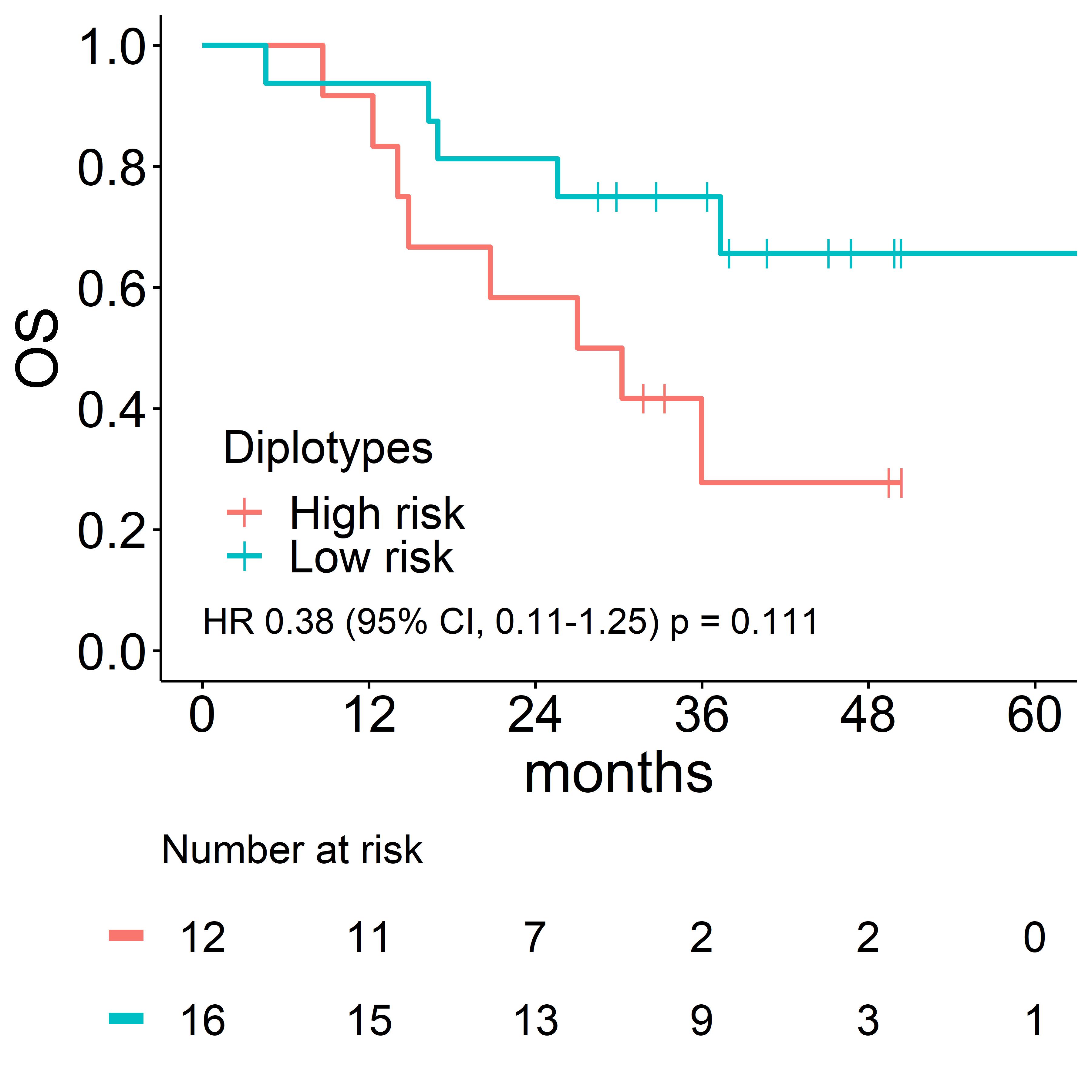
 (B)
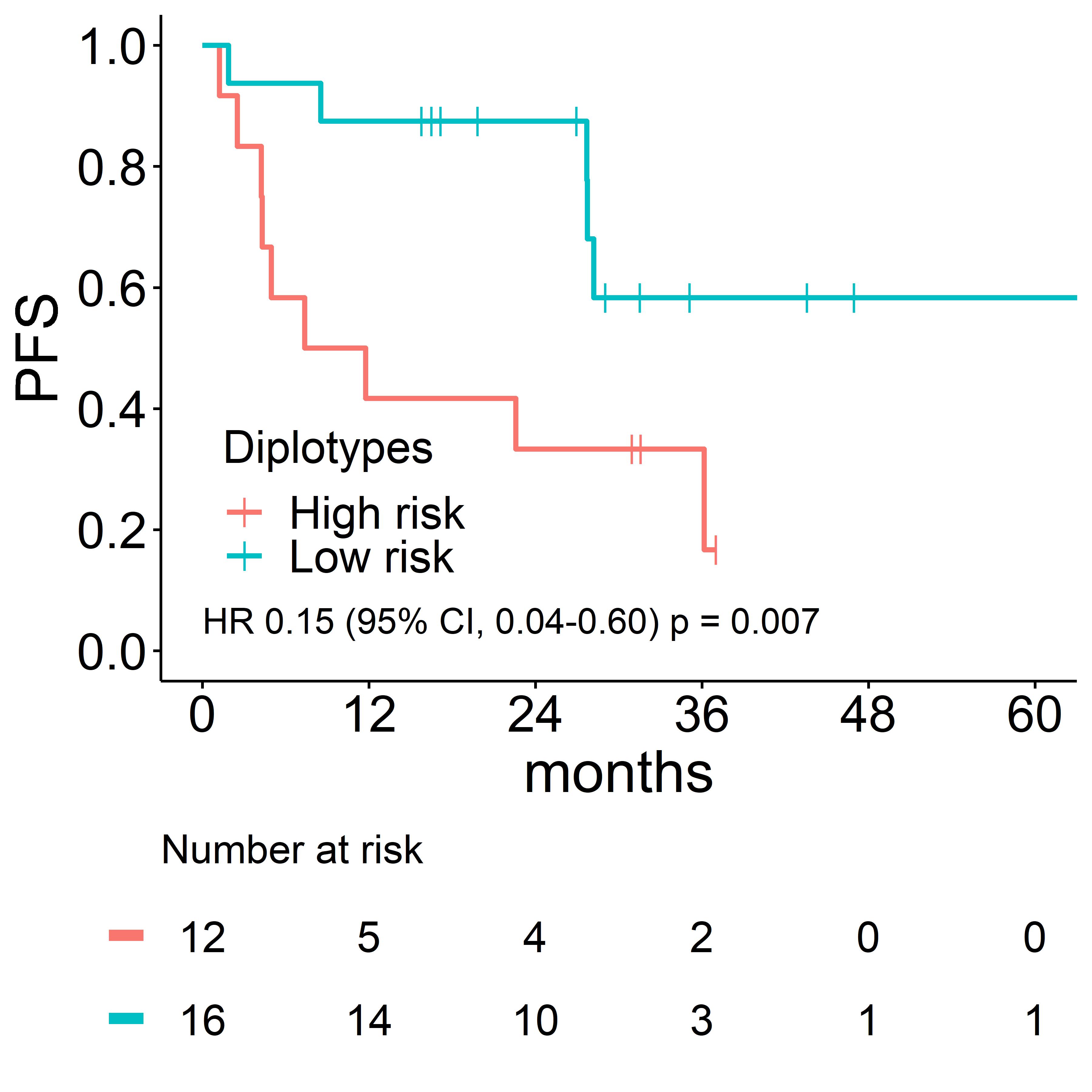


(C)
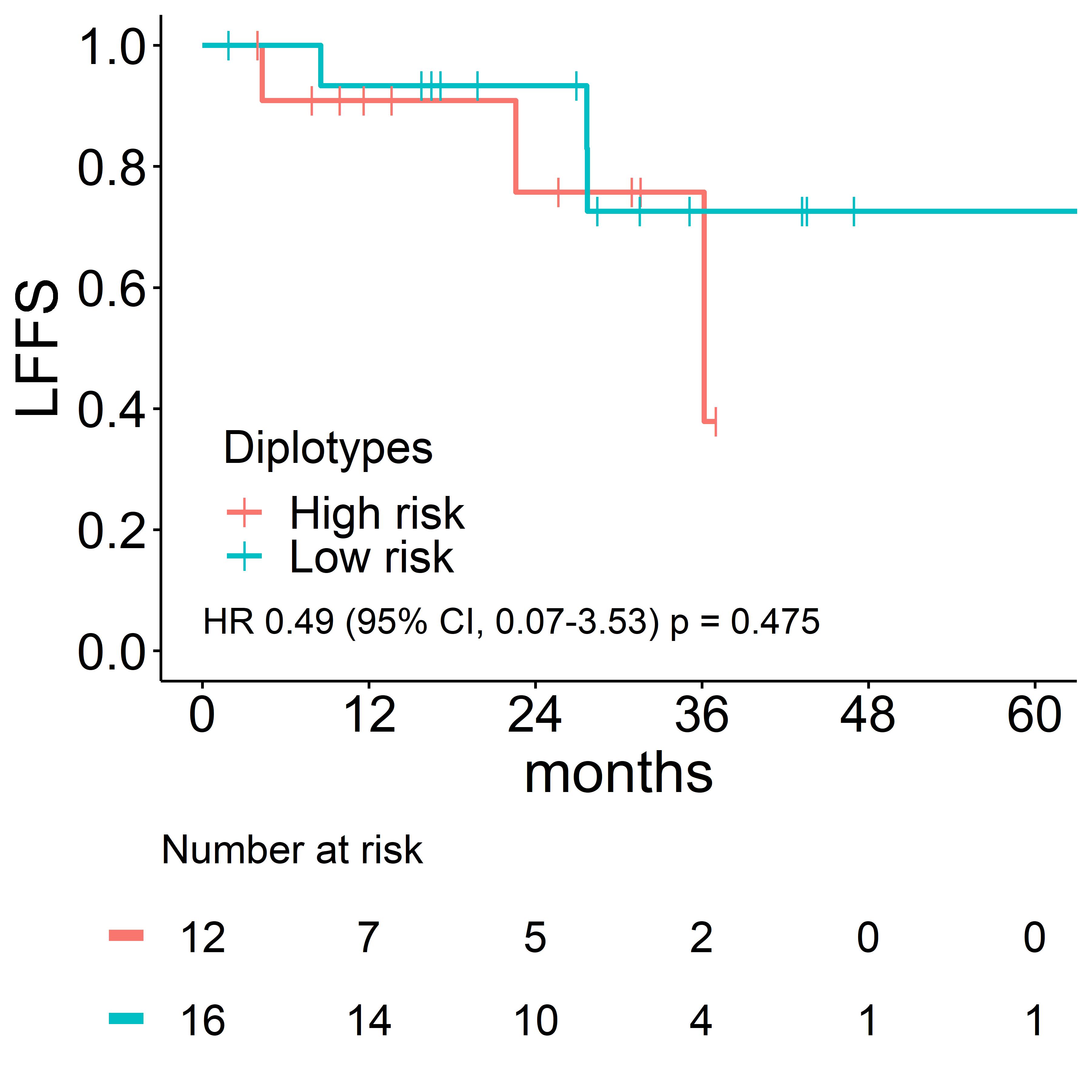
 (D)
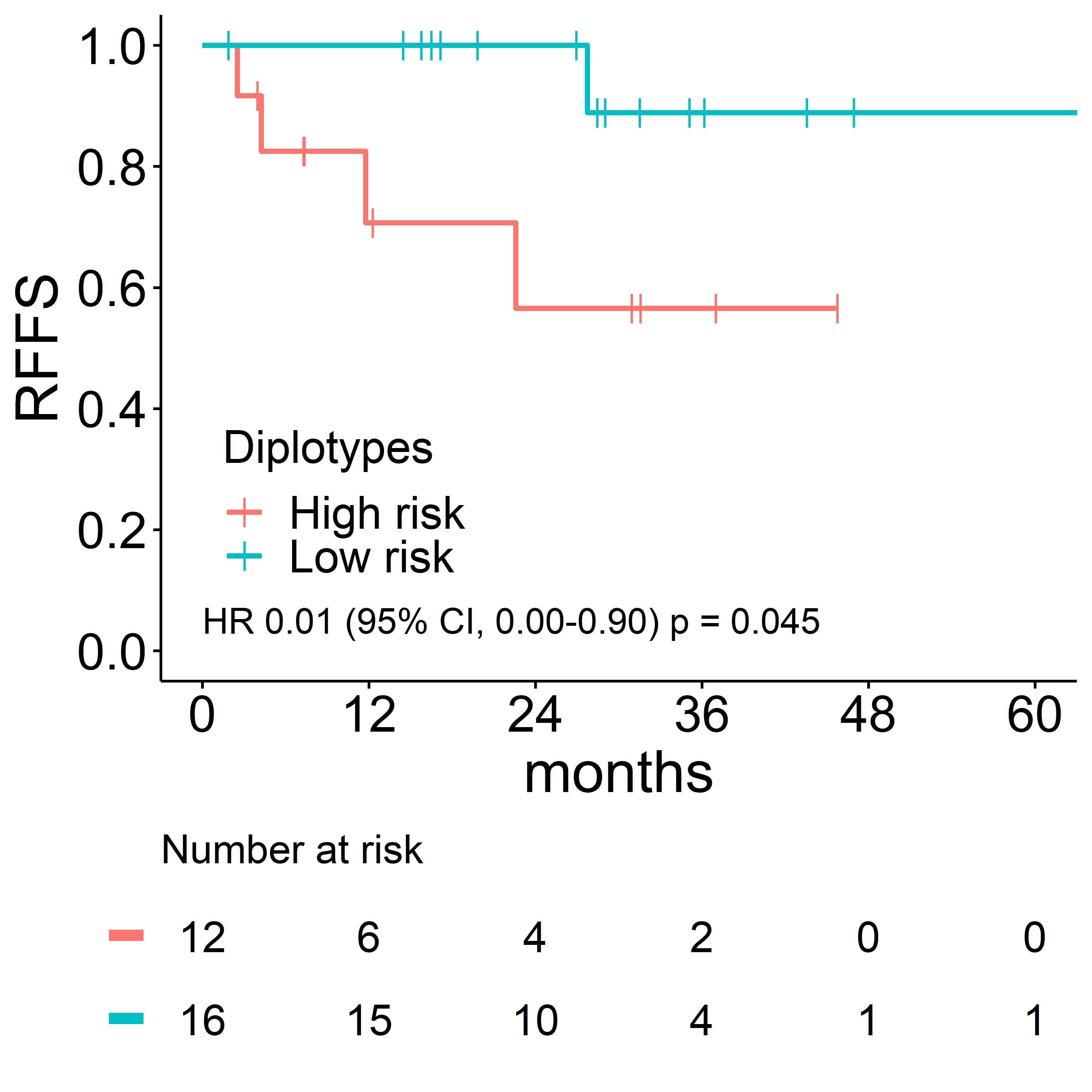


(E)
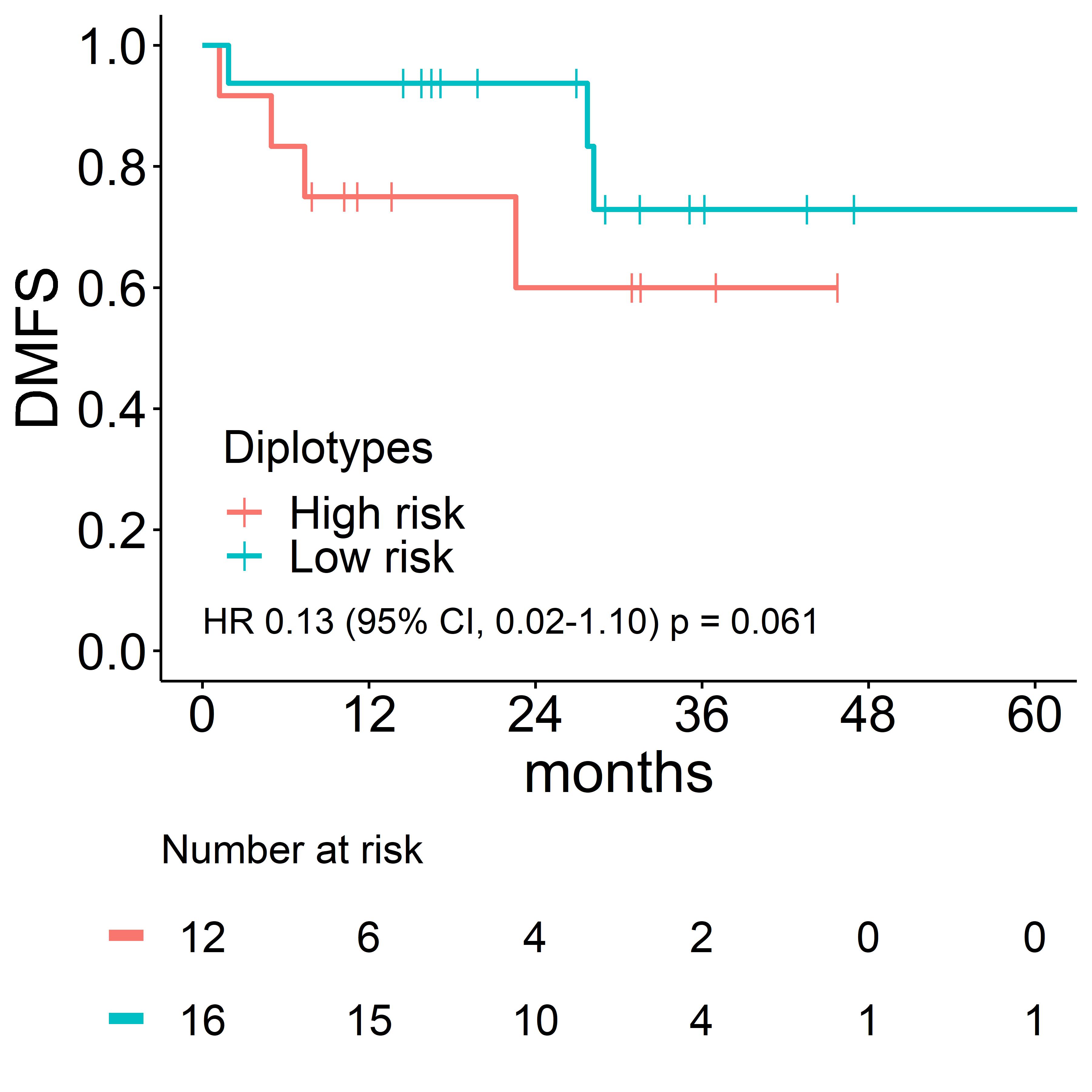


### **Figure S2.** Survival curves according to the risk groups of diplotypes of rs822336G>C-rs822337T>A-rs822338C>T in 96 patients treated with non-SBRT.

(A)
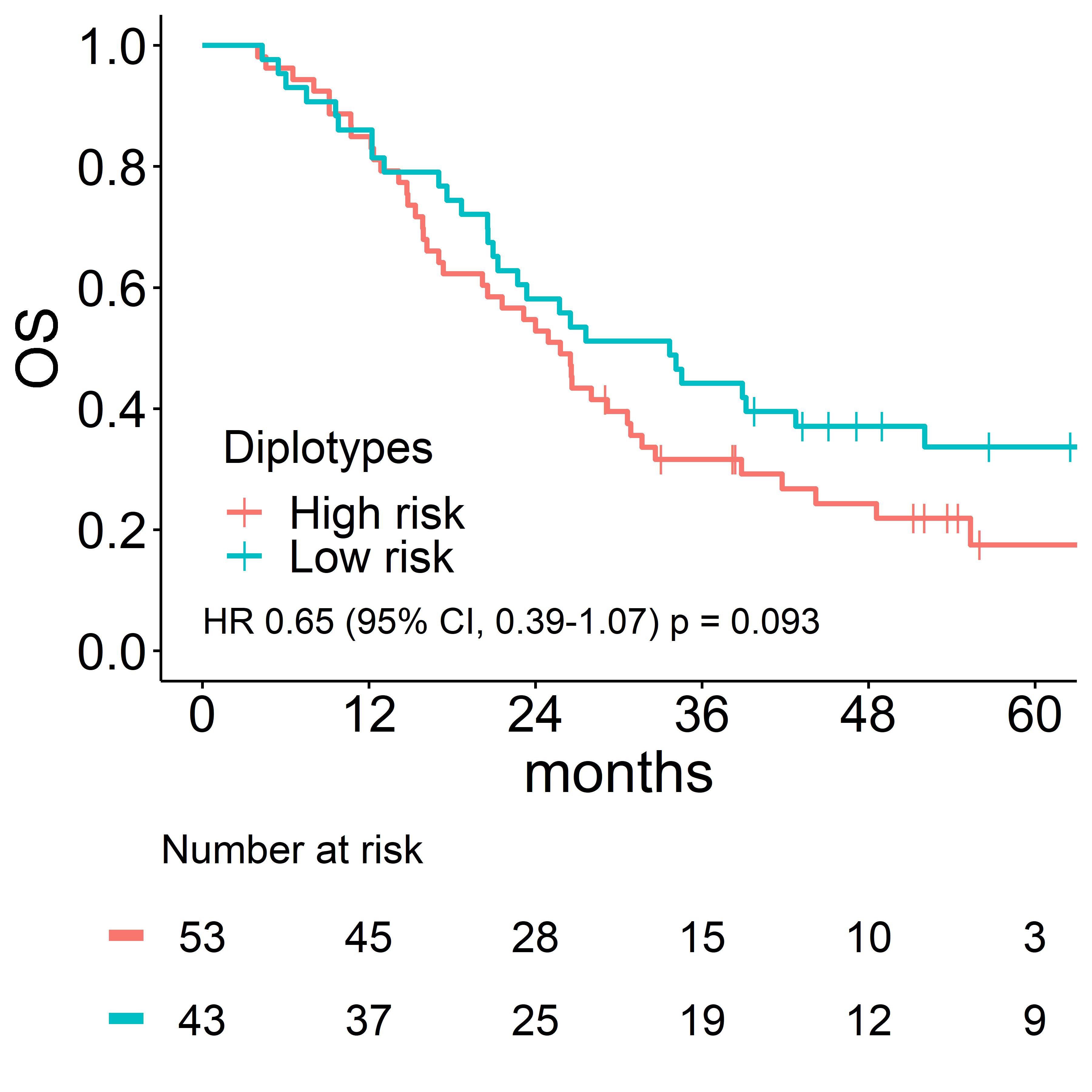
 (B)
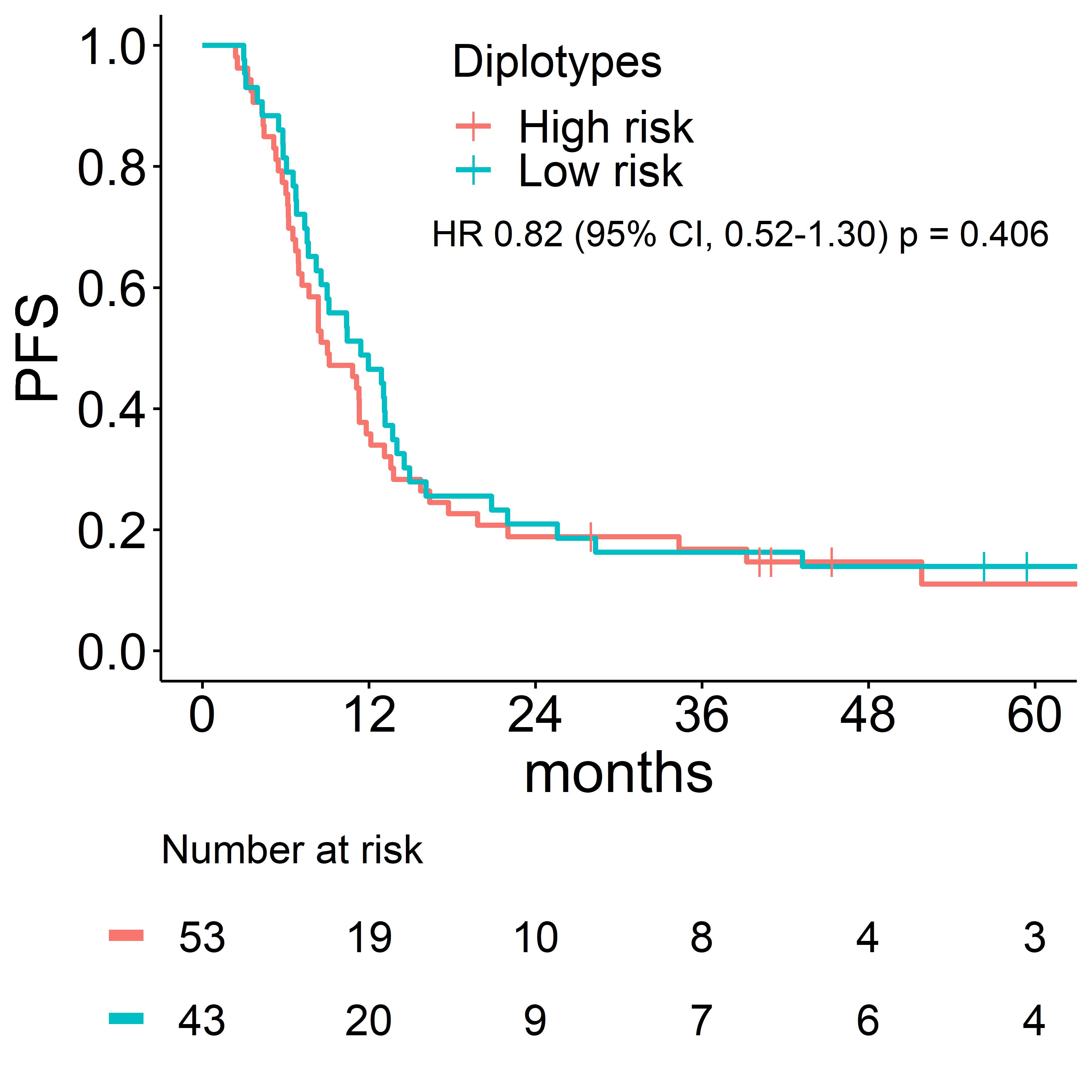


(C)
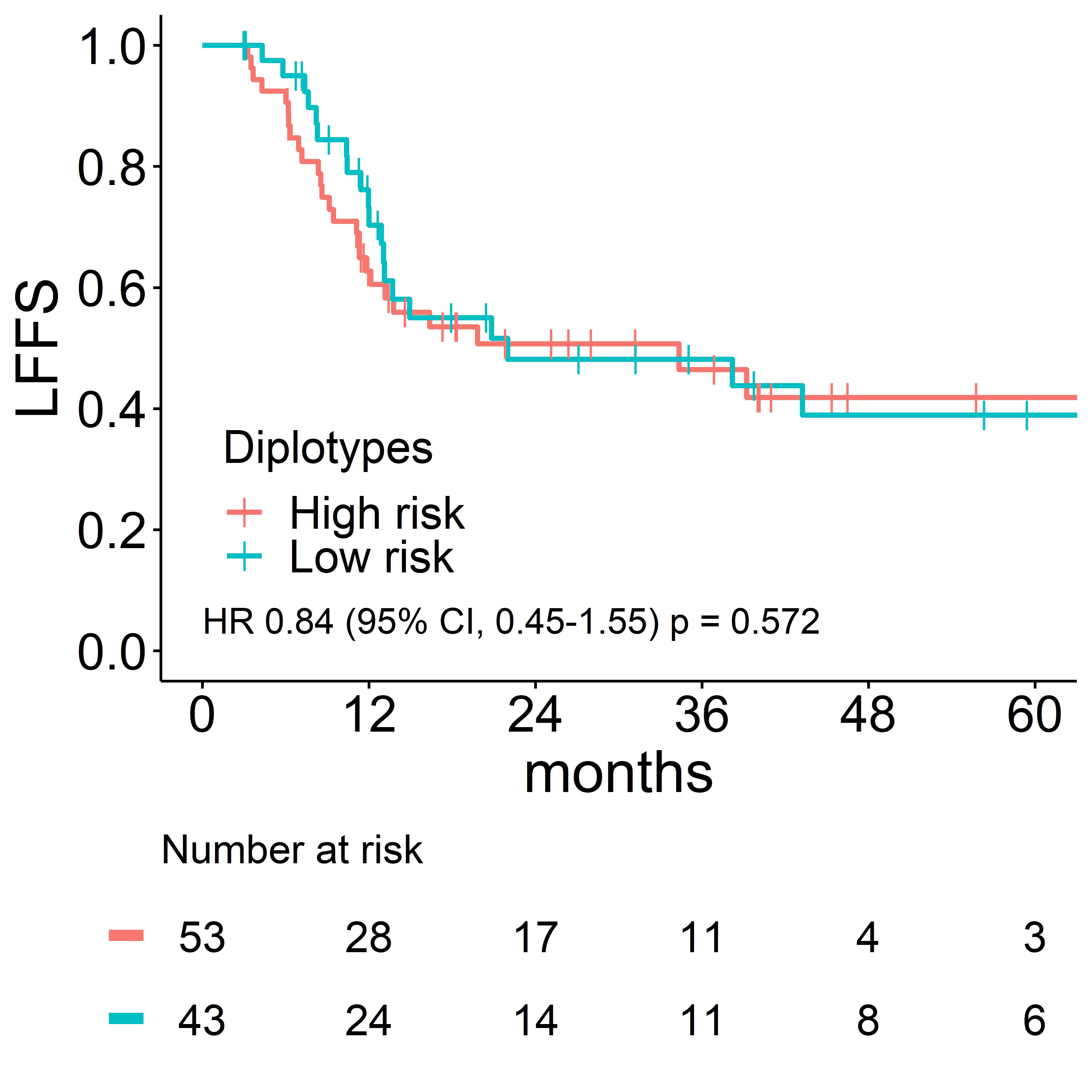
 (D)
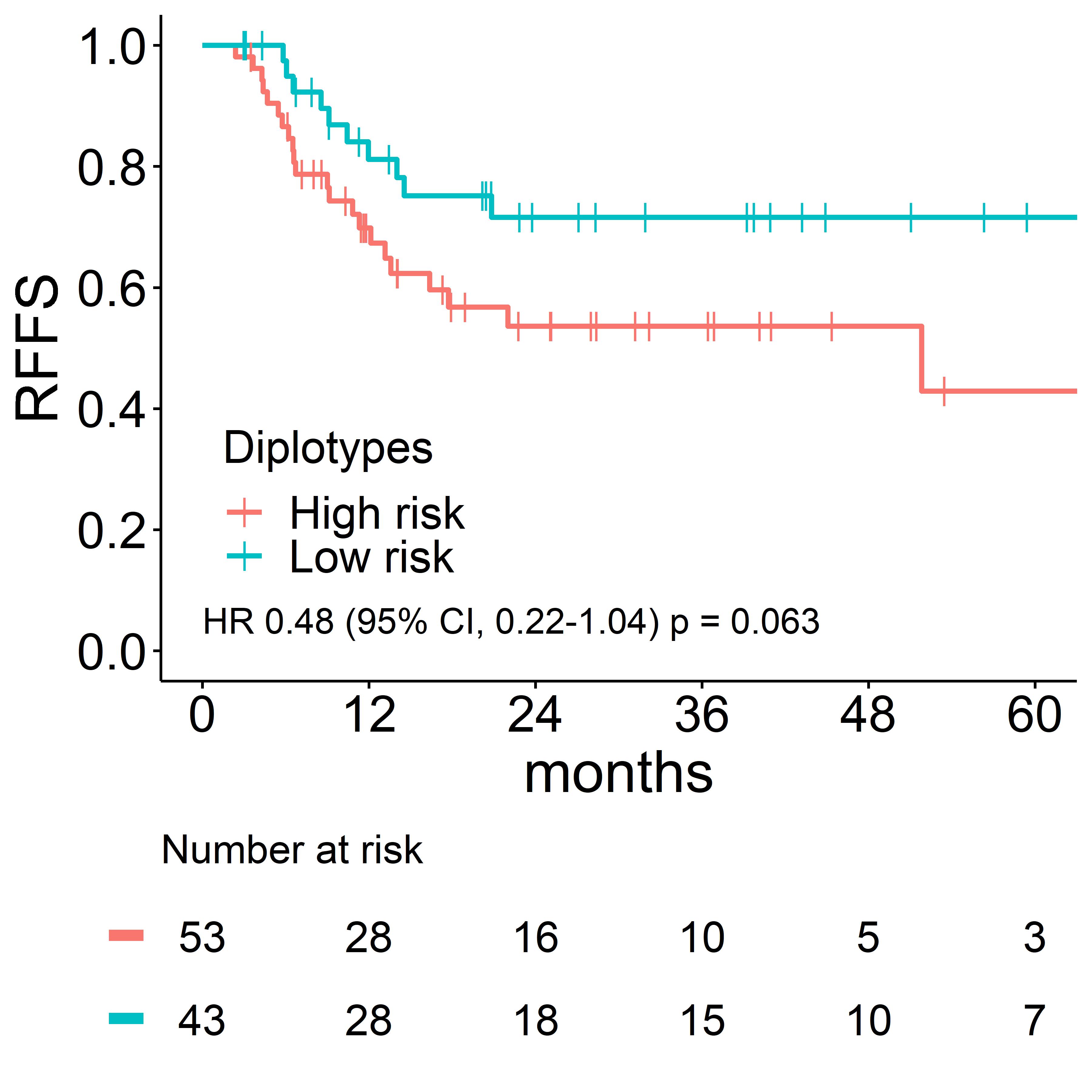


(E)
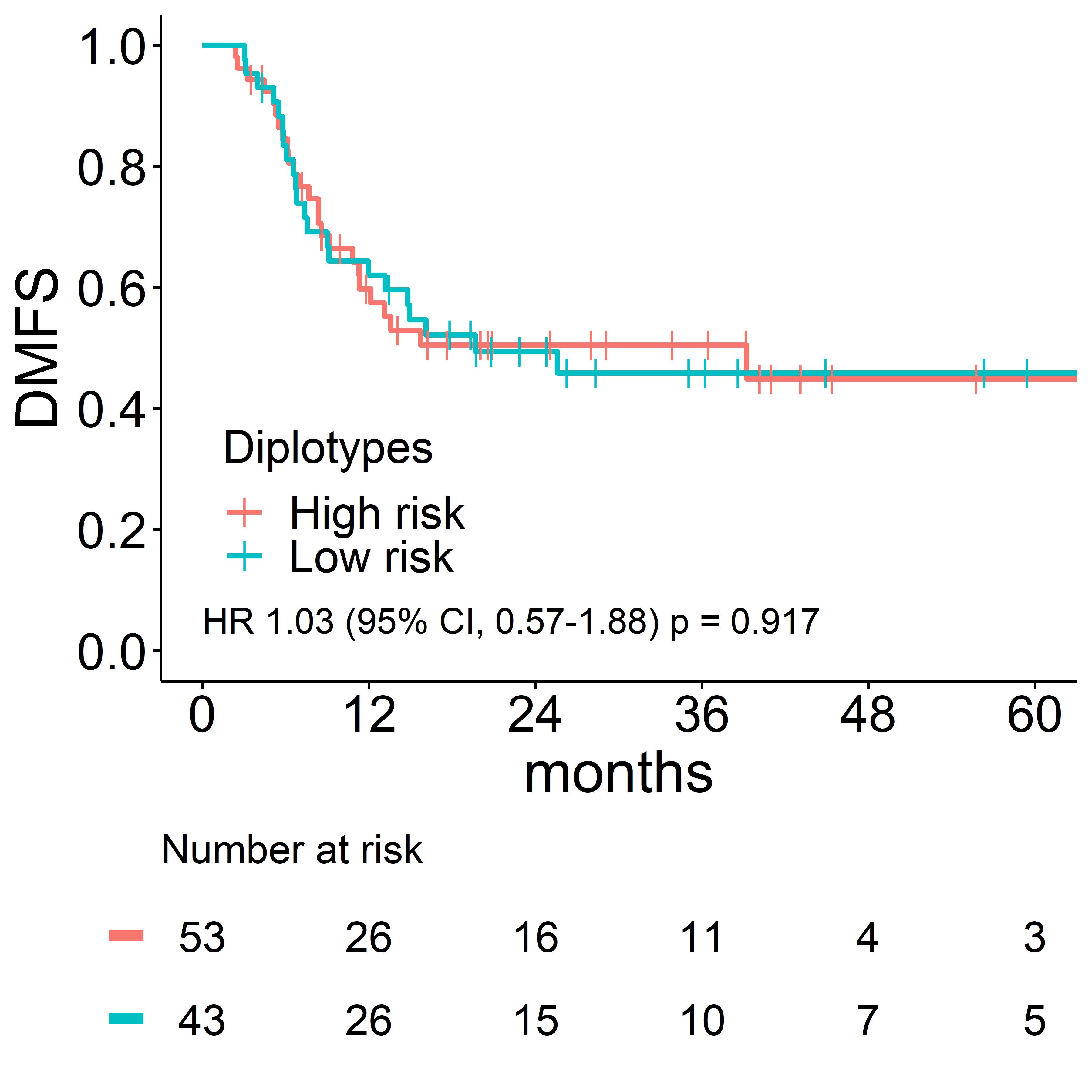

Supplement: Supplementary file 1 — Supplementary Material [file CAM4-10-8071-s001.docx]
